# How did Latinxs near the U.S.-Mexico border fare during the COVID-19 pandemic? A snapshot of anxiety, depression, and posttraumatic stress symptoms

**DOI:** 10.3389/fpsyg.2023.1241603

**Published:** 2023-08-17

**Authors:** Bianca T. Villalobos, Juventino Hernandez Rodriguez

**Affiliations:** Department of Psychological Science, The University of Texas Rio Grande Valley, Edinburg, TX, United States

**Keywords:** COVID-19, Latinx, anxiety, depression, posttraumatic stress

## Abstract

**Introduction:**

The current study documented levels of anxiety, depression, posttraumatic stress, and COVID-19 fears and impacts among Latinxs living near the U.S.-Mexico border during the COVID-19 pandemic.

**Methods:**

Participants of this cross-sectional study were 305 Latinx adults living in the Rio Grande Valley (RGV) who completed an online survey between June and November 2020.

**Results:**

About half of participants scored above the cut-off for anxiety (50.2%; GAD-7 scores ≥10) and depression (48.8%; PHQ-9 scores ≥10), and more than a quarter of participants showed clinical levels of posttraumatic stress (27.3%; PCL-5 scores ≥31). Latinxs reported on average 22 types of negative pandemic life impacts on the Epidemic-Pandemic Impacts Inventory. Endorsement of mental health symptoms, severity of COVID-19 fears, and COVID-related life impacts varied based on several demographic characteristics including gender, marital status, educational attainment, employment, income, insurance coverage, vulnerability to COVID-19, and essential worker status.

**Discussion:**

Overall, the cross-sectional results of this study revealed that RGV Latinx residents experienced high levels of psychological distress during the pandemic. Results suggest that Latinx women were most affected by the psychological consequences of the pandemic. More research is needed with communities living near the U.S.-Mexico border as they may be particularly vulnerable to mental health problems during the pandemic.

## 1. Introduction

The coronavirus (COVID-19) pandemic has changed the lives of people all over the world. Subsequently, these life changes have resulted in additional stressors and heightened levels of mental health symptoms for communities of color in the United States (U.S.). However, data on the mental health status of Latinx communities during the pandemic is limited. The purpose of this study is to provide a snapshot of the mental health status of Latinx adults living near the U.S.-Mexico border. We examined prevalence rates of anxiety, depression, and posttraumatic stress (PTS) symptomology, the severity and impairment of COVID-19 fears, and COVID-19-related life impacts. We also examined whether mental health symptoms and COVID-19 fears and impacts varied by specific psychosocial demographic variables.

Evidence suggests that Latinxs, among other communities of color, were disproportionally impacted by the COVID-19 pandemic. The Color of Coronavirus Project, an ongoing study that examines U.S. COVID-19-related deaths by race and ethnicity, found that the age-adjusted mortality rates for Latinxs were three times higher than for non-Latinx White people (Gawthrop, 2022). In addition to higher mortality rates, preexisting inequities in social determinants of health exacerbated the impact of the pandemic on Latinxs (Fortuna et al., 2020). For example, pre-pandemic Latinxs had lower wages, lower income, and less access to employment benefits and health care compared to non-Latinx White people (Gould et al., 2020). These long standing disparities were highlighted by the economic challenges faced by almost half of Latinxs (49%) who reported they or someone in their household had a reduction in pay or lost their job due to the pandemic (Krogstad et al., 2020). Moreover, risk of exposure to the virus could have been higher for Latinxs because, compared to non-Latinx White people, they were less likely to hold occupations that gave them flexibility to work remotely and engage in social distancing (Gould et al., 2020).

Latinx low-income immigrants have been disproportionately impacted by pandemic-related stressors due to high rates of poverty, structural barriers to receive social services, and limited access to healthcare (Clark et al., 2020; Fortuna et al., 2020). Many Latinx immigrant families experience an abundance of fear and worry due to the larger sociopolitical context that may directly impact their livelihood. Undocumented Latinx immigrants and asylum seekers are at particular risk of mental health consequences due to their histories of traumatic stress coupled with poor conditions at shelters and detention centers (Garcini et al., 2020; Mercado et al., 2022). Moreover, anti-immigration policies and rhetoric have created fear and mistrust in state and federal institutions, including mental health agencies (Garcini et al., 2020). Latinxs living near the U.S.-Mexico border may also experience significant pandemic-related stressors. For example, the Rio Grande Valley (RGV), a collection of four counties at the southernmost tip of Texas, contributed to 10.8% of coronavirus-related deaths in the state in 2020 even though it only represented 4.7% of Texas’ population (Texas Department of State Health Services, 2023). A significant proportion of RGV residents live in poverty, with estimates ranging between 25.6 and 34.2% (U.S. Census Bureau, 2020). High rates of COVID-19 diagnoses and mortality, coupled with pre-existing structural barriers, likely lead RGV residents and similar border communities to experience distress surrounding the pandemic. Indeed, this trend of worry about contracting the virus, accessing basic needs, and barriers to health care are experienced in many Latinx communities (American Psychological Association, 2020).

The data available on the impact of the COVID-19 pandemic on Latinx mental health is limited. Although several nationally representative studies have examined the mental health impact of the COVID-19 pandemic on adults (i.e., Holingue et al., 2020; McGinty et al., 2020; Twenge and Joiner, 2020; Warren et al., 2021), they are limited in their ability to describe Latinx-specific responses to the pandemic. For example, Latinx ethnicity was a protective factor against psychological distress in the study by Holingue et al. (2020) while it was a risk factor in the study by McGinty et al. (2020). Though these findings could help us understand how Latinxs’ psychological distress compared to the general population, the Latinx representation in these studies (7.5–14.7%) was modest. Additionally, these studies did not explore specific Latinxs subgroups that may be at particular risk. Recent studies have identified factors that could be associated with mental health symptoms in Latinxs. Using data from a nationally representative sample of Latinxs, Gomez-Aguinaga et al. (2021) found that Latinas, compared to Latinos, were more likely to report mental health symptoms. Additionally, results revealed that participants who knew undocumented immigrants were more likely to report mental health issues, compared to participants who did not know undocumented immigrants. Although useful, this study did not focus on specific mental health problems.

The purpose of this study is to address the need for more research on specific Latinx communities by providing descriptive information about the frequency and severity of psychological distress among South Texas Latinxs living near the U.S.-Mexico border during the outset of the COVID-19 pandemic. The specific aims of the current study were (1) to report prevalence rates of anxiety, depression, posttraumatic stress, illness and virus fears, and life changes during the pandemic, and (2) to identify demographic characteristics related to mental health symptoms, illness and virus fears, and life changes.

## 2. Materials and methods

### 2.1. Procedure

The study was posted online via Qualtrics as an anonymous online survey and was available in Spanish and English. Participants were eligible if they were at least 18 years of age, resided in the U.S., and identified as Latinx, Latina, Latino, or Hispanic. Participants were recruited through social media (e.g., Twitter, Facebook), a national organization (e.g., National Latinx Psychological Association) and university listservs. Data collection lasted approximately 4 months (June 24–November 2, 2020), and participants who finished the survey were given the option to enter a raffle to win a gift card. For the current study, the analytical sample was a subset of 363 participants located in four counties that comprise the Rio Grande Valley of South Texas: Cameron, Hidalgo, Starr, and Willacy. Residents in these four counties are primarily Latinx with percentages of Hispanic/Latino ethnicity ranging from 88.1 to 96.3% (U.S. Census Bureau, 2021). The sample was further reduced to 305 for whom a total score or subscale score could be computed on at least one of the dependent variables. All procedures were approved by the Institutional Review Board at the University of Texas Rio Grande Valley.

### 2.2. Measures

In addition to demographic information, participants completed a variety of mental health screening questionnaires and newly developed COVID-19 measures. Participants were also asked to indicate whether they or an individual living in the home was an essential worker or vulnerable to COVID-19. Regarding essential worker status, we defined this term for participants using the U.S. Department of Homeland Security Cybersecurity and Infrastructure Security Agency’s (CISA) list of essential work sectors (Version 1.0; issued March 2020):

Health care employees, first responders, food and agriculture employees, energy employees, water and sanitation, transportation and logistics, public workers, manufacturing, communications and information technology, community-based government operations and essential functions, financial services, hazardous material management, defense industrial base, and chemical management employees.

We defined vulnerability to COVID-19 using the CDC’s initial list of populations at high risk for severe illness from the virus, which was expanded in June 2020 to include other groups not listed here:

People aged 65 and older, people living in a nursing home or long-term care facility, people with chronic lung disease or moderate to severe asthma, people with serious heart conditions, people who are immunocompromised, people with severe obesity, people with diabetes, people with chronic kidney disease undergoing dialysis, and people with liver disease.

#### 2.2.1. Anxiety and depression

Patient Health Questionnaire (PHQ) modules on anxiety [Generalized Anxiety Disorder 7-item Scale (GAD-7); Spitzer et al., 2006] and depression [PHQ 9-item Scale (PHQ-9); Kroenke et al., 2001] were used to assess anxiety and depression symptom severity. Items from the GAD-7 and PHQ-9 were developed based on the DSM-IV, but closely follow existing diagnostic criteria from the DSM-5. Participants respond to items by indicating the frequency they were bothered by symptoms in the prior 2 weeks on a Likert scale from 0 (Not at all) to 3 (Nearly every day). Recommended cut points for total severity scores on the PHQ-9 distinguish between those with mild (5), moderate (10), moderately severe (15), and severe (20) depression. Severity cut points for mild, moderate, and severe anxiety on the GAD-7 are 5, 10, and 15, respectively. For both the GAD-7 and PHQ-9, scores of 10 or higher suggest a clinical diagnosis might be warranted (Kroenke et al., 2001; Spitzer et al., 2006).

A study examining the psychometric properties of the GAD-7 with a sizable Latinx community sample reported excellent internal consistency (*α* = 0.93) for the overall sample, high reliability for English (*α* = 0.91) and Spanish (*α* = 0.94) language-preference groups, and convergent validity with a measure of perceived stress (Mills et al., 2014). Similarly, studies examining the PHQ-9 with Latinx adults reported strong psychometric properties, with good internal consistency (*α* = 0.84–0.85) and construct validity (Huang et al., 2006; Merz et al., 2011). In this study, internal consistency was excellent for the PHQ-9 (*α* = 0.91) and GAD-7 (*α* = 0.94).

#### 2.2.2. Posttraumatic stress

The PTSD Checklist for DSM-5 (PCL-5) (Weathers et al., 2013) is a 20-item self-report measure that assesses PTS symptoms. Participants are asked how often they were bothered by each symptom in the last month on a Likert scale from 0 (Not at all) to 4 (Extremely). A cut-point of 31 to 33 can be used to determine a provisional diagnosis of PTSD and a referral to PTSD treatment. Previous studies have reported strong psychometric properties for the PCL (Blevins et al., 2015; Bovin et al., 2016). To our knowledge, there are no studies assessing the psychometric properties of the PCL-5 with Spanish-speaking populations; however, studies have found the Spanish and English PCL-Civilian (PCL-C) versions for the DSM-IV were equivalent in terms of differential item functioning (Miles et al., 2008).

For this study, we adapted the PCL-5 to specifically ask participants about symptoms in response to the COVID-19 pandemic. Items with the words “stressful experience” were adapted to state “coronavirus” to directly assess reactions to the pandemic. Other studies have used this procedure using the PCL-5 in pandemic-related research (e.g., Liu N. et al., 2020). In the current study, analyses used the total severity score, which had excellent internal consistency (*α* = 0.94).

#### 2.2.3. Psychological impact of COVID-19

The Fear of Illness and Virus Evaluation (FIVE) (Sáez-Clarke et al., 2022) is a 35-item self-report measure that assesses COVID-19 pandemic fears. The measure reports on four subscales: fears about contamination and illness (9 items), fears about social distancing (9 items), behaviors related to illness and virus fears (13 items), and impact of illness and virus fears (2 items). The first two subscales are scored from 1 (I am not afraid of this at all) to 4 (I am afraid of this all of the time). The behavior subscale is scored from 1 (I have not done this in the last week) to 4 (I did this all the time last week). Lastly, the two impact items are scored from 1 (Not true for me at all) to 4 (Definitely true). A composite fear score can be calculated by summing items from the first two subscales (Sáez-Clarke et al., 2022). A study examining the psychometric properties of the FIVE among a non-clinical sample of Chilean adults found good to excellent internal consistency across subscales (*α* = 0.81–0.91) (Cottin et al., 2021). Convergent validity was supported by comparing FIVE scores to depressive and posttraumatic stress symptoms.

In this study, internal consistency was excellent for fears related to contamination and illness (*α* = 0.92), fears related to social distancing (*α* = 0.91), and the composite fear score (*α* = 0.94). Internal consistency for the subscales of behaviors related to illness and virus fears (*α* = 0.74) and impact (*α* = 0.80) was acceptable and good, respectively. All four subscales and the composite fear score were used in analyses.

#### 2.2.4. COVID-19 impacts

COVID-19 pandemic impacts were measured with the 92-item Epidemic-Pandemic Impacts Inventory (EPII) (Grasso et al., 2020). Participants were asked to identify ways in which the pandemic has impacted various aspects of their life and the lives of people in their home. The measure assesses 10 domains of impact: work and employment (11 items), education and training (2 items), home life (13 items), social activities (10 items), economic (5 items), emotional health and wellbeing (8 items), physical health problems (8 items), physical distancing and quarantine (8 items), infection history (8 items), and positive change (19 items). For each item, participants were provided the following response options: “Yes, Me,” “Yes, Person in Home,” “No,” and “Not Applicable.” In this study, we collapsed participants’ first two responses (i.e., “Yes, Me” or “Yes, Person in Home”) into one category and combined the last two responses (i.e., “No” or “Not Applicable”) into another category. A cumulative negative change score was calculated by summing all domains except for the positive change domain. The cumulative negative change score and the positive change domain were used in analyses.

### 2.3. Statistical analyses

Statistical analyses were performed using IBM SPSS, Version 28. Bivariate and point-biserial correlations were conducted to examine the relations among all study variables (see Supplementary Table S1). A series of independent-samples *t-*tests and one-way ANOVAs were conducted to examine whether mental health variables (anxiety, depression, and PTS severity) and COVID-19 related variables (psychological impact and number of life impact counts) differed by participant demographic variables: gender, language, nativity, education level, employment status, essential worker in household, vulnerable person in household, income, and insurance status. All independent-samples *t-*test statistics were obtained assuming unequal variances. Welch’s *F*-test is reported for all ANOVAs and post-hoc comparisons were computed using the Games-Howell correction. Effect sizes are reported using Hedges’ correction. Because of the number of analyses run, values of *p* less than 0.01 were regarded as significant for independent-samples *t-*tests and one-way ANOVAs.

#### 2.3.1. Missing data

Missing data varied for the dependent variables: 0.3–3.6% for the EPII subscales; 7.5–9.5% for the FIVE subscales; 9.8% for the GAD-7; 10.5% for the PHQ-9; 12.5% for the PCL-5. Approximately 18.3% of participants did not provide a valid response to an item about race. Most of these individuals instead reported their ethnicity or nationality, which was already captured by the inclusion criteria of the survey (e.g., “Hispanic,” Chicana,” and “Mexican-American”). Thus, analyses did not include race. All other demographic variables had less than 3.5% missing data.

## 3. Results

### 3.1. Demographic characteristics of the sample

Participant demographics are provided in Table 1. Of the 305 Latinx participants, the majority identified as female (79.3%), White (77.7%), U.S.-born (85.2%), and single (67.2%). The average age was about 28 years (range = 18–69). Participants were from four counties in the lower Rio Grande Valley, near the U.S.-Mexico border: Hidalgo (62.0%), Cameron (34.1%), Starr (2.0%), and Willacy (2.0%). Most participants reported English was their primary language (77.7%), had at least a college degree (54.0%), an annual household income of at least $40,000 (52.4%), and were insured (63.0%). Regarding employment status, 46.6% were students and 31.5% worked full-time. In addition, 66.6% indicated they or someone in the home was an essential worker, and 59.6% indicated they or someone in the home was vulnerable to COVID-19. On average, participants had approximately four people living in their household.

**Table 1:** Participant demographic characteristics.

Variable	*N* = 305	
	*n* (%)	*M* (*SD*)
Gender		
Female	242 (79.3)	
Male	53 (17.4)	
Gender non-conforming/Non-binary	7 (2.3)	
Transgender female	3 (1.0)	
Age		
In years		27.96 (11.47)
Primary language		
English	237 (77.7)	
Spanish	65 (21.3)	
Other (English and Spanish equally)	3 (1.0)	
Race		
White	237 (77.7)	
Black or African-American	3 (1.0)	
American Indian/Alaska Native	2 (0.7)	
Asian	1 (0.3)	
Other	1 (0.3)	
Multiracial	5 (1.6)	
Not reported	56 (18.4)	
Birth country		
United States	259 (85.2)	
Mexico	36 (11.8)	
Puerto Rico	2 (0.7)	
Venezuela	1 (0.3)	
Spain	1 (0.3)	
Cuba	1 (0.3)	
Other country, unknown	4 (1.3)	
If foreign-born, years lived in the United States		18.23 (13.16)
Marital status		
Single, never married	205 (67.2)	
Married	66 (21.6)	
Living with partner	15 (4.9)	
Divorced, widowed, separated	19 (6.2)	
Education		
High school or GED	54 (17.7)	
Technical training/certificate	8 (2.6)	
Some college	78 (25.6)	
Associate’s degree	59 (19.3)	
Bachelor’s degree	72 (23.6)	
Master’s degree	23 (7.5)	
Doctoral/Professional degree	11 (3.6)	
Employment status		
Full-time	96 (31.5)	
Part-time	23 (7.5)	
Student	142 (46.6)	
Not working (homemaker, unemployed, disabled)	31 (10.2)	
Furloughed or laid off	13 (4.3)	
Essential worker in the home		
Yes	199 (66.6)	
No	100 (33.4)	
Person vulnerable to COVID-19 in the home		
Yes	180 (59.6)	
No	122 (40.4)	
Income		
<$40,000	140 (47.6)	
$40,000–80,000	94 (32.0)	
>$80,000	60 (20.4)	
Health insurance		
Yes	191 (63.0)	
No	112 (37.0)	
Household size		
Number of people in household (range = 1–9)		3.86 (1.52)

### 3.2. Descriptive statistics on mental health and COVID-19 variables

See Table 2 for means, standard deviations, and percentages for each of the mental health and COVID-19 measures. Utilizing recommended cut-points for the GAD-7 and PHQ-9 (scores ≥10), about half of participants had moderate to severe anxiety (50.2%) and depression (48.8%). After examination of scores at or above the cut-point of 15, about a quarter of individuals would benefit from active psychotherapy treatment for symptoms of anxiety (27.3%) and depression (27.2%). Lastly, using the PCL-5’s recommended cut-point, a quarter of individuals (27.3%) demonstrated PTS symptoms congruent with a provisional diagnosis of PTSD.

**Table 2:** Mean total scores, standard deviations, and range of scores on measures.

Measure	*M* (*SD*)	Range	*n* (%)
GAD-7	9.85 (6.81)	0–21	
None/Minimal (score 0–8)			76 (27.6)
Mild (score 5–9)			61 (22.2)
Moderate (score 10–14)			63 (22.9)
Severe (score 15–21)			75 (27.3)
PHQ-9	10.41 (7.55)	0–27	
None minimal (score 0–8)			68 (24.9)
Mild (score 5–9)			72 (26.4)
Moderate (score 10–14)			59 (21.6)
Moderately Severe (score 15–19)			31 (11.4)
Severe (score 20–27)			43 (15.8)
PCL-5	20.43 (17.08)	0–68	
Subclinical symptoms (score < 31)			194 (72.7)
PTSD provisional diagnosis (score ≥ 31)			73 (27.3)
FIVE			
Composite fear	46.37 (13.96)	19–76	
Contamination/Illness fear	23.70 (7.23)	9–36	
Social distancing fear	22.76 (8.18)	10–40	
Behaviors	40.33 (6.87)	23–56	
Impact	4.98 (2.17)	2–8	
EPII			
Work/Employment	3.67 (2.21)	0–11	
Education/Training	0.81 (0.71)	0–2	
Home life	2.07 (2.08)	0–12	
Social activities	5.65 (2.14)	0–10	
Economic impact	0.66 (0.91)	0–5	
Emotional wellness/Wellbeing	3.47 (1.86)	0–8	
Physical health	3.17 (1.53)	0–7	
Physical distancing/Quarantine	2.41 (1.80)	0–8	
Infection history	0.68 (0.92)	0–4	
Negative impacts (all domains above)	22.35 (8.38)	1–43	
Positive changes	8.64 (3.81)	0–18	

GAD-7, Generalized Anxiety Disorder Scale; PHQ-9, Patient Health Questionnaire; PCL-5, PTSD Checklist; FIVE, Fear of Illness and Virus Evaluation; EPII, Epidemic Pandemic Impacts Inventory.

Participants also reported on their fears related to the virus, social distancing, safety behaviors, and impairment related to the coronavirus. Participants had on average 22 negative and nine positive life impacts because of the coronavirus pandemic (see Table 3 for the most common impacts reported). Spending more time on screens and devices, more time sitting down or being sedentary, and family celebrations getting canceled or restricted were the most common negative impacts. The most common positive changes included being more appreciative of things usually taken for granted, paying more attention to one’s personal health, and more quality time with family or friends in person or from a distance.

**Table 3:** Frequencies and percentages of common negative and positive impacts regarding COVID-19.

EPII impacts	n (%)
Negative	
More time sitting down or being sedentary	270 (90.3)
Spent more time on screens and devices	269 (90.0)
Family celebrations canceled or restricted	272 (89.5)
Unable to do enjoyable activities or hobbies	252 (82.9)
Planned travel or vacations canceled	236 (77.9)
Separated from family/close friends	231 (75.7)
Less physical activity or exercise	218 (73.6)
Increase in sleep problems or poor sleep quality	220 (73.3)
Increase in mental health problems or symptoms	216 (72.5)
Overeating or eating more unhealthy foods	211 (71.0)
Unable to participate in social clubs, sports teams, or usual volunteer activities	212 (70.0)
Spent a lot of time disinfecting at home due to close contact with people who might be infected at work	198 (65.1)
Had to continue to work even though in close contact with people who might be infected	187 (61.7)
Religious or spiritual activities canceled or restricted	179 (58.7)
Increase in workload or work responsibilities	168 (55.3)
Isolated or quarantined due to possible exposure to this disease	160 (54.1)
Had a child in home who could not go to school	159 (52.1)
Limited physical closeness with child or loved one due to concerns of infection	139 (47.1)
Reduced work hours or furloughed	133 (44.3)
Isolated due to existing health conditions that increase risk of infection or disease	126 (43.2)
Hard time making the transition to working from home	118 (38.7)
Unable to attend in-person funeral for family/friend who died	113 (37.4)
Positive	
More appreciative of things usually taken for granted	237 (80.3)
Paid more attention to personal health	229 (78.4)
More quality time with family/friends in person or from a distance	212 (71.9)
More time doing enjoyable activities	195 (66.3)
Developed new hobbies or activities	163 (55.4)
Paid more attention to preventing physical injuries	162 (55.3)
Improved relationships with family/friends	162 (54.9)
Ate healthier foods	159 (54.3)
Found greater meaning in work, employment, or school	148 (50.3)

*N* = 305; endorsement of negative impacts and positive changes due to COVID-19 are not mutually exclusive. EPII, Epidemic Pandemic Impacts Inventory.

### 3.3. Demographic characteristics associated with mental health symptom severity

See Table 4 for mental health measure means by demographic variables. Compared to males, females had significantly higher average anxiety, *t*(69.47) = −3.11, *p* = 0.003, *d* = 0.51, depression, *t*(74.92) = −3.45, *p* < 0.001, *d* = 0.52, and PTS scores, *t*(78.85) = −3.17, *p* = 0.002, *d* = 0.47. No other group differences were found for anxiety and PTS.

**Table 4:** Mental health mean total scores (GAD-7, PHQ-9, and PCL-5) by demographics.

Characteristic	Anxiety	Depression	Posttraumatic stress
Gender			
Female	10.42 (6.64)^a^	11.00 (7.48)^a^	21.80 (17.30)^a^
Male	6.96 (7.19)^b^**	7.14 (6.99)^b^***	13.90 (15.36)^b^**
Primary language			
English	9.67 (6.80)	9.85 (7.47)	19.06 (15.65)
Spanish	10.38 (6.84)	12.29 (7.50)	25.07 (20.51)
Nativity			
U.S-born	10.01 (6.85)	10.48 (767)	20.51 (17.07)
Foreign-born	8.87 (6.62)	9.97 (6.88)	19.95 (17.36)
Marital status			
Single, never married	10.39 (7.04)	11.88 (7.90)^a^	21.37 (17.37)
Married or living with partner	8.31 (6.15)	6.89 (5.46)^b^***	16.75 (14.58)
Divorced, widowed, separated	10.83 (6.47)	10.44 (6.86)	26.17 (21.34)
Education			
Less than college degree	10.19 (6.99)	11.32 (7.62)^b^***	20.70 (16.56)
Undergraduate degree	10.12 (6.63)	10.47 (7.58)^b^**	21.57 (17.81)
Graduate degree	7.33 (6.50)	6.47 (5.84)^a^	15.00 (15.77)
Employment status			
Full-time	9.00 (6.35)	7.88 (6.76)^a^	17.99 (16.71)
Part-time	9.68 (7.32)	7.91 (6.00)	14.14 (11.64)
Student	10.12 (6.70)	12.14 (7.67)^b^***	21.91 (17.36)
Not working	10.93 (7.84)	11.75 (7.85)	24.31 (18.29)
Essential worker in the home			
No	8.63 (6.49)	9.29 (6.78)	17.67 (16.14)
Yes	10.41 (6.97)	10.93 (7.83)	21.97 (17.47)
Vulnerable person in the home			
No	8.63 (6.97)	9.32 (7.81)	17.49 (16.81)
Yes	10.70 (6.64)	11.11 (7.21)	22.27 (16.74)
Income			
<$40,000	10.26 (6.81)	11.95 (7.95)^a^	21.71 (18.06)
$40,000–80,000	10.03 (6.60)	9.92 (6.63)	19.63 (15.40)
>$80,000	7.93 (6.57)	7.42 (6.68)^b^***	16.67 (15.32)
Health insurance			
No	10.39 (6.89)	12.12 (7.61)^a^	21.75 (17.75)
Yes	9.44 (6.74)	9.31 (7.30)^b^**	19.37 (16.51)

Superscripts indicate significant differences between groups. **p < 0.01, ***p < 0.001.

In addition to gender, there were several demographic differences in depression. Significant group differences in depression were found by participant marital status, *F*(2, 46.77) = 16.61, *p* < 0.001. Single participants had higher average depression compared to those who were married or cohabitating (*p* < 0.001, *d* = 0.69). Significant group differences in depression by educational attainment were also found, *F*(2, 91.01) = 7.46, *p* < 0.001. Compared to those with graduate or professional degrees, participants with less than a college level of education (*p* < 0.001, *d* = 0.66), and those with undergraduate degrees (*p* = 0.007, *d* = 0.55), reported higher depression. Employment status also mattered, *F*(2, 74.76) = 7.43, *p* < 0.001, such that participants who worked full-time had lower depression scores compared to students (*p* < 0.001, *d* = 0.58). Significant group differences were found based on annual household income for depression, *F*(2, 142.07) = 7.61, *p* < 0.001. Those with incomes below $40,000 had higher average depression compared to those with incomes above $80,000 (*p* < 0.001, *d* = 0.60). Finally, uninsured participants had significantly higher average depression scores compared to those with health insurance, *t*(197.35) = 2.97, *p* = 0.003, *d* = 0.38.

### 3.4. Demographic characteristics associated with COVID-19 fears

See Table 5 for mean scores on the FIVE subscales by demographic variables. Female participants had significantly higher mean scores compared to males for: Composite Fear, *t*(70.04) = −2.70, *p* = 0.009, *d* = 0.47; Behaviors Related to Illness and Virus Fears, *t*(71.58) = −3.81, *p* < 0.001, *d* = 0.62; and Impact of Illness and Virus Fears, *t*(78.95) = −3.00, *p* = 0.004, *d* = 0.45. Participants who had a vulnerable person at home had significantly higher mean scores compared to those who did not have a person vulnerable to COVID-19: Composite Fear, *t*(244.11) = −3.13, *p* = 0.001, *d* = 0.40; and Impact of Illness and Virus Fears, *t*(239.28) = −2.92, *p* = 0.004, *d* = 0.36. Analyses also revealed a significant group difference in FIVE scores by income on: Fears about Social Distancing, *F*(2, 143.21) = 4.95, *p* = 0.008; and Impact of Illness and Virus Fears, *F*(2, 138.80) = 3.63, *p* = 0.029. Regarding insurance status, participants who were insured had statistically significant higher mean scores compared to those without health insurance on the Fears of Social Distancing subscale, *t*(206.13) = 3.06, *p* = 0.002, *d* =0.38.

**Table 5:** Fear of illness and virus evaluation mean total scores by demographics.

Characteristic	Composite fear	Fears of contamination and illness	Fears of social distancing	Behaviors	Impact
Gender					
Female	47.63 (13.54)^a^	24.32 (7.08)^a^	23.41 (8.02)	41.24 (6.55)^a^	5.15 (2.16)^a^
Male	41.43 (15.03)^b**^	21.27 (7.51)^b^**	20.20 (8.65)	37.18 (7.05)^b^***	4.20 (2.02)^b^**
Primary language					
English	46.12 (14.15)	23.62 (7.37)	22.62 (8.30)	40.20 (6.71)	4.96 (2.15)
Spanish	57.63 (13.38)	24.27 (6.79)	23.36 (7.86)	40.53 (7.40)	5.05 (2.23)
Nativity					
U.S-born	46.72 (13.84)	23.87 (7.21)	22.90 (8.06)	40.43 (6.76)	5.07 (2.16)
Foreign-born	44.16 (14.69)	22.54 (7.42)	21.90 (8.99)	39.66 (7.60)	4.40 (2.15)
Marital status					
Single	47.40 (14.21)	23.93 (7.29)	23.54 (8.28)	40.24 (6.94)	5.09 (2.17)
Married or living with partner	43.28 (13.39)	22.61 (6.88)	20.67 (7.97)	40.00 (6.59)	4.49 (2.11)
Divorced, widowed, separated	48.94 (12.13)	25.74 (7.75)	23.61 (6.78)	42.61 (7.30)	5.78 (2.07)
Education					
Less than college degree	45.98 (14.59)	23.64 (7.84)	22.54 (8.30)	39.30 (7.10)	5.07 (2.02)
Undergraduate degree	47.79 (13.72)	24.15 (6.97)	23.63 (8.13)	41.22 (6.66)	4.97 (2.29)
Graduate degree	42.27 (11.41)	22.10 (5.23)	20.17 (7.53)	41.00 (6.37)	4.60 (2.28)
Employment status					
Full-time	45.72 (13.58)	23.89 (6.86)	21.94 (8.12)	41.22 (6.68)	4.81 (2.20)
Part-time	46.23 (13.74)	22.77 (7.23)	23.46 (8.37)	40.68 (6.69)	5.09 (2.20)
Student	46.17 (14.17)	23.38 (7.22)	22.88 (8.40)	39.90 (6.99)	5.13 (2.08)
Not working	48.53 (14.48)	24.76 (8.13)	23.78 (7.64)	39.58 (7.05)	4.78 (2.37)
Essential worker in the home					
No	46.09 (14.12)	23.45 (7.33)	22.68 (8.34)	40.32 (7.06)	5.14 (2.09)
Yes	46.40 (13.97)	23.72 (7.24)	22.79 (8.13)	40.35 (6.82)	4.85 (2.19)
Vulnerable person in the home					
No	43.01 (13.62)^a^	22.09 (7.43)^a^	21.03 (7.78)^a^	40.15 (6.69)	4.51 (2.17)^a^
Yes	48.54 (13.68)^b**^	24.71 (6.88)^b^**	23.91 (8.21)^b^**	40.45 (6.99)	5.28 (2.11)^b^**
Income					
<$40,000	47.54 (14.24)	24.25 (7.43)	23.31 (8.13)	40.35 (7.17)	5.23 (2.17)
$40,000–80,000	46.33 (14.81)	23.02 (7.32)	23.42 (8.74)	39.91 (6.59)	4.92 (2.19)
>$80,000	42.80 (11.73)	23.21 (6.65)	19.80 (7.15)	40.93 (6.60)	4.32 (2.01)
Health insurance					
No	48.58 (14.60)	23.99 (7.70)	24.68 (8.23)^a^	39.96 (7.34)	5.03 (2.23)
Yes	44.89 (13.37)	23.40 (6.90)	21.57 (7.97)^b^**	40.56 (6.62)	4.91 (2.12)

Superscripts indicate significant differences between groups. **p < 0.01, ***p < 0.001.

### 3.5. Demographic characteristics associated with positive and negative COVID-19 impacts

See Table 6 for count scores on the Positive Changes and Negative Impacts subscales of the EPII by demographic groups. Females reported significantly more negative changes, *t*(73.40) = −4.17, *p* < 0.001, *d* = 0.64, compared to males. Lastly, participants who reported they had an essential worker or vulnerable person living in the home had significantly more negative impacts than individuals who did not have an essential worker, *t*(233.67) = −3.59, *p* < 0.001, *d* = 0.41, or vulnerable person at home, *t*(249.92) = −3.63, *p* < 0.001, *d* = 0.43.

**Table 6:** Epidemic pandemic impacts inventory negative and positive impact counts by demographics.

Characteristic	Negative impacts	Positive change
Gender		
Female	23.29 (8.17)^a^	8.51 (3.57)
Male	18.02 (8.32)^b^***	9.71 (4.40)
Primary language		
English	21.99 (8.55)	8.62 (3.83)
Spanish	23.48 (7.76)	8.65 (3.70)
Nativity		
U.S-born	22.81 (8.49)	8.59 (3.89)
Foreign-born	19.71 (7.37)	8.93 (3.41)
Marital status		
Single	22.42 (8.27)	8.33 (3.87)
Married or living with partner	21.52 (8.72)	9.30 (3.85)
Divorced, widowed, separated	25.16 (7.86)	9.05 (2.61)
Education		
Less than college degree	22.42 (8.37)	8.17 (3.82)
Undergraduate degree	23.28 (8.14)	8.87 (3.76)
Graduate degree	18.50 (8.55)	9.67 (3.81)
Employment status		
Full-time	20.95 (8.39)	9.14 (3.65)
Part-time	23.48 (8.89)	9.46 (3.49)
Student	22.55 (7.99)	8.29 (3.79)
Not working	24.18 (9.12)	8.32 (4.29)
Essential worker in the home		
No	20.10 (7.21)^a^	9.07 (3.56)
Yes	23.53 (8.80)^b^***	8.53 (3.91)
Vulnerable person in the home		
No	20.21 (8.52)^a^	8.76 (3.52)
Yes	23.77 (8.04)^b^***	8.53 (4.01)
Income		
<$40,000	22.49 (8.39)	8.22 (3.75)
$400,000–800,000	22.16 (7.81)	8.84 (3.63)
>$80,000	21.85 (8.76)	9.90 (3.82)
Health insurance		
No	23.32 (8.18)	8.23 (3.90)
Yes	21.70 (8.46)	8.92 (3.74)

Superscripts indicate significant differences between groups. ***p < 0.001.

## 4. Discussion

This study helps fill a gap in the literature by providing results on the mental health status of Latinx adults living near the U.S.-Mexico border during the outset of the pandemic. Results corroborate what many commentaries in the field have posited regarding the potential impact of COVID-19 on Latinxs (e.g., Fortuna et al., 2020; Lund, 2020; Thakur et al., 2020). Indeed, a considerable amount of Latinxs reported struggling with clinically significant symptoms of anxiety, depression, and PTS. Almost half of participants had moderate to severe levels of distress related to anxiety (50.2%) and depression (48.8%). In addition, one in four individuals in this study met the clinical cutoff for a provisional diagnosis of PTSD (27.3%), specifically in response to COVID-19 as an index trauma. Rates of anxiety (45.4%; GAD-7 scores ≥10) and depression (43.3%; PHQ-9 scores ≥10) were slightly higher, and rates of PTSD (31.8%; PCL-Civilian scores ≥45) were slightly lower than those reported by a majority non-Latinx White sample (Liu C.H. et al., 2020).

The current study also identified several key demographic characteristics associated with high levels of depression: female gender (including transgender females), single marital status, educational attainment less than a graduate degree, lack of health insurance, and an annual household income of less than $40,000. Conversely, only female gender was associated with anxiety and PTS symptom severity. The psychological impact of COVID-19 (i.e., FIVE scores) was greatest for Latinxs who identified as female, were vulnerable to COVID-19 (or living with a person vulnerable to COVID-19) or were uninsured. In addition, female gender, being or living with an essential worker at home, and being or living with a person vulnerable to COVID-19 were related to a greater number of negative life impacts. On the other hand, we found no demographic differences for positive life impacts.

Latinas were especially impacted by COVID-19 in our study. It is possible that gender role norms and the greater burden of household responsibilities placed on Latinas contributed to experiences of distress and few positive life changes during the pandemic. For example, Latinas may be juggling multiple demanding roles that compound pandemic-related stress, such as being a parent of a child going to school remotely, being a caregiver for ill family members, and being a worker, homemaker, or student. Moreover, Latinas had higher rates of unemployment immediately after the start of the pandemic given they were more likely to work in industries considered nonessential (Gould et al., 2020). Stress due to temporary or continued unemployment may have impacted Latinas’ mental health and heightened worries about loss of income for basic needs. Overall, Latinas may need more support and resources to manage the psychological impact of the pandemic.

Our findings run contrary to expectations that immigrant communities are at higher risk of the medical, economic, legal, and social consequences of the pandemic (Clark et al., 2020). In this study, foreign-born and primarily Spanish-speaking Latinxs were impacted similarly to US-born and English-speaking Latinxs. The pandemic is often described as a great equalizer, but it has also exposed inequities in our health care systems. It is possible that Latinxs living in border communities, regardless of nativity and language, were similarly impacted by the pandemic and disparities exist in comparison to non-Latinx White populations. Previous research shows Latinxs in general encountered barriers to accessing culturally- and linguistically-congruent resources to help them navigate the risk of virus exposure and treatment (Bigelow et al., 2021), possibly explaining high rates of anxiety, depression and PTS symptoms. It is also possible that particular immigrant Latinx groups, such as undocumented adults and DACAmented students, are at higher risk for experiencing the consequences of the pandemic compared to immigrant Latinxs with documentation status. Unfortunately, we did not collect data on documentation status in this study.

Findings suggest several indicators of low socioeconomic status (SES) were related to higher levels of depression: less education, student status, single (never married) status, lower annual household income, and lack of health insurance. Taken together, these indicators are likely tied to the experiences of college students. It is likely these Latinx college students represented another vulnerable group during the pandemic because of lack of financial resources, mandated stay-at-home orders, social distancing, and reliance on remote learning. Students likely experienced high levels of depression due to lack of engagement in pleasurable activities, lack of social contact with peers, and disruptions in the progress of their academic goals.

Upon further examination of the data, we found many single individuals lived with their parents (79.0%) and had a person vulnerable to COVID-19 in the home (62.6%). Therefore, it is also possible that levels of depression may be tied to experiences of grief due to loss of loved ones affected by COVID-19 or are tied to caregiving of sick older adults in the home. Likewise, Latinxs with lower incomes might report more depression due to the potential loss of income resulting from mandated social distancing or sickness. Low SES Latinxs may be at particular risk of depression because they lack financial resources to manage the negative life impacts of the pandemic.

In addition, vulnerability to COVID-19 was one of the few demographic variables related to the FIVE. People who were vulnerable or living with family members at risk of experiencing complications due to COVID-19 were afraid between “some” or “most” of the time about contamination issues including hospitalization, their own death, or that a family member or friend could get sick and die. Individuals with vulnerability to COVID-19, or lived with someone vulnerable to COVID-19, showed more frequent fears of social distancing including being afraid of losing their jobs, of not being able to celebrate events due to illness, and of not having enough food or money to pay bills. Interestingly, the FIVE helped us identify that differences in fear levels did not necessarily translate to differences in engaging in safety behaviors like use of sanitizer, wearing masks, avoiding people, wiping surfaces, or checking the internet for virus updates. Individuals with or without someone vulnerable in the home engaged in these behaviors to the same degree (about most of the time in the previous week). In addition, those with someone vulnerable to COVID-19 at home had about three and a half more negative life impacts than those without a vulnerable person at home, revealing the disproportionate consequences of the pandemic for those with chronic conditions. Although vulnerability to COVID-19 was not identified as a risk factor for anxiety or depression, COVID-19 fears or the number of negative life impacts may mediate this relation.

Another main finding from the study was that Latinxs reported more negative life impacts if they were an essential worker or living with an essential worker in the home. Negative life impacts for these individuals might be related to increases in workload or responsibilities. While essential workers were at increased risk of contracting the virus given their employment in critical sectors of the workforce, their fears about COVID-19 were no different from their counterparts. This group of Latinxs may, out of necessity, have developed cognitive coping strategies that attenuated fears of COVID-19 to perform their job duties in the face of negative life consequences. Given the lack of hospital beds in the Rio Grande Valley, essential workers in the health care sector may have especially been burdened by the soaring infection and hospitalization rates along under-resourced areas of the border. For example, cross-border traffic can place these regions at risk of high transmission of communicable diseases, a situation that is compounded by shortages of health care providers in many border and rural regions like those in this study (Texas Department of State and Health Services, 2021).

### 4.1. Limitations and future directions

The findings of the current study should be considered in light of limitations. Given the purpose of the study was to provide a snapshot of the state of Latinxs’ mental health during the COVID-19 pandemic, the results are limited by the cross-sectional design of the study. Despite the cross-sectional nature of the study, there was evidence to suggest a rise in sleep and mental health problems since almost three in four participants reported that they experienced an increase since the start of the pandemic. The severity and frequency of mental health symptoms likely fluctuated with the country’s containment of the virus, increased or laxed restrictions in social distancing, mandates on wearing masks, and resulting consequences. Second, data were primarily obtained from a convenience sample, which can limit generalizability due to participants self-selecting to complete the online survey. Ultimately, our results might reflect a group of individuals who are more concerned about their mental health and COVID-19’s impact on their lives. Although we collected data from an online convenience sample, mental health screening measures administered in the study were well-validated and included cut points to indicate a probable clinical diagnosis of depression, anxiety, and PTSD. Third, it is possible that these results may only generalize to Latinxs living near the U.S.-Mexico border as we did not conduct comparative analyses with other Latinxs living in other parts of the U.S. Although living near the border is a unique experience, it is possible that these findings may be applicable to Latinxs like those captured in this study, namely low-income Latina students and single adults. Another limitation is that the sample was mostly educated with a scarcity of male and LGBTQ+ Latinxs. Since most residents in Texas report Mexican ancestry, results may also not represent the mental health adjustment of other Latinx subgroups. The study also did not assess preexisting mental health symptoms or psychiatric diagnoses before the start of the pandemic, which makes it difficult to determine whether reported mental health symptoms represent an exacerbation of symptoms or are a direct result of the pandemic.

Future studies should employ longitudinal methods to analyze the long-term mental health impact of COVID-19 on Latinxs living in the U.S. even now that the virus is contained, and vaccinations have been distributed to vulnerable individuals and essential workers. Our study primarily focused on the onset of the pandemic in the U.S. and Latinxs living near the U.S.-Mexico border. Therefore, there is still a need to learn more about how mental health rates have changed over time for all Latinxs living across the U.S. Researchers can also identify whether other subgroups of Latinxs (e.g., South Americans, Puerto Ricans) are more susceptible to the psychological consequences of the pandemic. Additionally, it would be helpful for researchers and clinicians to assess mental health symptoms with this population using structured interviews. This is now feasible, compared to the start of the pandemic, because clinics have had considerable experience using telehealth and most have resumed in-person appointments. Given that one in four individuals had a probable diagnosis of PTSD due to COVID-19, existing PTSD measures should now include the pandemic as a potential index trauma, like natural disasters.

Assessment of additional risk or protective factors may help elucidate findings, such as adherence to traditional Latinx cultural values. Latinxs who are less acculturated may endorse stronger cultural values, such as fatalism and spiritualism, that could serve as protective factors against COVID-19 fears. For instance, placing one’s life in the hands of a higher power might reduce worry about illness, but may also result in fewer preventative safety behaviors (i.e., wearing masks and social distancing). Subsequent research on Latinxs should analyze rates of mental health service utilization and barriers to access to care during the coronavirus, including use of telemental health services for the treatment of anxiety, depression, and PTSD. Given the border regions included in this study are all designated health professional shortage areas, future studies can identify how mental health can be impacted by the availability of health and mental health care resources at rural border regions compared to resources in metropolitan and non-border regions. Identifying border residents as particularly vulnerable during public health crises can help with allocation of healthcare resources and additional funding.

### 4.2. Conclusion

Within the first 7 months after shelter-in-place orders were announced in the U.S., Latinxs in this study reported numerous negative changes in several life domains (e.g., employment, home life, social activities, emotional wellness, and physical health). Indeed, our study found a substantial proportion of Latinxs reported high levels of anxiety, depression, and PTS that would warrant treatment. Despite limitations, this study provides preliminary evidence of psychological distress among Latinxs living near the U.S.-Mexico border during the COVID-19 pandemic and highlighted potential risk factors for further examination. Our study also increases the data available for Latinxs who make up only small proportions of existing national surveys. Future studies are needed to track changes in mental health after the pandemic as they relate to variables that are known to contribute to mental health disparities for Latinxs.

## Data availability statement

The raw data supporting the conclusions of this article will be made available by the authors, without undue reservation.

## Ethics statement

The study involving humans were approved by the University of Texas Rio Grande Valley Institutional Review Board. The study was conducted in accordance with the local legislation and institutional requirements. The ethics committee/institutional review board waived the requirement of written informed consent for participation from the participants or the participants’ legal guardians/next of kin because the study was determined to be exempt involving only anonymous participation in an online survey.

## Author contributions

BV and JH contributed to conception and design of the study. BV organized the database, performed the statistical analysis, and wrote the first draft of the manuscript. JH wrote sections of the manuscript. All authors contributed to manuscript revision, read, and approved the submitted version.

## Conflict of interest

The authors declare that the research was conducted in the absence of any commercial or financial relationships that could be construed as a potential conflict of interest.

## Publisher’s note

All claims expressed in this article are solely those of the authors and do not necessarily represent those of their affiliated organizations, or those of the publisher, the editors and the reviewers. Any product that may be evaluated in this article, or claim that may be made by its manufacturer, is not guaranteed or endorsed by the publisher.
