# Supplementary material for: How did Latinxs near the U.S.-Mexico border fare during the COVID-19 pandemic? A snapshot of anxiety, depression, and posttraumatic stress symptoms
Source: Front Psychol. 2023 Aug 17;14:1241603. doi: 10.3389/fpsyg.2023.1241603 (PMC10471481; doi:10.3389/fpsyg.2023.1241603)
Supplement: Supplementary file 1 [file Data_Sheet_1.docx]

***Supplementary Material***

**How Did Latinxs Near the U.S.-Mexico Border Fare During the COVID-19 Pandemic? A Snapshot of Anxiety, Depression, and Posttraumatic Stress Symptoms**

**Bianca T. Villalobos^1^*, Juventino Hernandez Rodriguez^1^**

***Correspondence:** [bianca.villalobos@utrgv.edu](mailto:bianca.villalobos@utrgv.edu)

**Supplementary Table 1**

*Bivariate and Point-biserial Correlations Among Study Variables*

| Variable | 1 | 2 | 3 | 4 | 5 | 6 | 7 | 8 | 9 |
| --- | --- | --- | --- | --- | --- | --- | --- | --- | --- |
| 1. Gender | – |  |  |  |  |  |  |  |  |
| 2. Language | -.03 | – |  |  |  |  |  |  |  |
| 3. Nativity | .07 | .33*** | – |  |  |  |  |  |  |
| 4. Marital Status | -.02 | .08 | -.02 | – |  |  |  |  |  |
| 5. Education | .06 | .10 | .02 | .41*** | – |  |  |  |  |
| 6. Essential | .05 | -.01 | .05 | .01 | -.02 | – |  |  |  |
| 7. Vulnerable | -.02 | -.01 | .06 | -.09 | -.04 | .14* | – |  |  |
| 8. Income | -.09 | .16** | .00 | .31*** | .27*** | .11 | -.02 | – |  |
| 9. Insurance | .01 | .14* | .12* | .24*** | .17** | .04 | -.07 | .27*** | – |
| 10. GAD-7 | .20** | -.04 | .06 | -.14* | -.08 | .12* | .15* | -.11 | -.07 |
| 11. PHQ-9 | .20** | -.13* | .02 | -.29*** | -.20*** | .10 | .12 | -.22*** | -.18** |
| 12. PCL-5 | .18** | -.15* | .01 | -.13* | -.06 | .12 | .14* | -.11 | -.07 |
| 13. FIVE - Contamination Fear | .17** | -.04 | .06 | -.09 | -.01 | .02 | .18** | -.07 | -.04 |
| 14. FIVE - Social Distancing Fear | .15* | -.04 | .04 | -.16** | -.04 | .01 | .18** | -.18** | -.18** |
| 15. FIVE - Behaviors | .24*** | -.02 | .04 | -.03 | .17** | .00 | .02 | .05 | .04 |
| 16. FIVE - Impact | .17** | -.02 | .11 | -.14* | -.03 | -.07 | .18** | -.16* | -.03 |
| 17. FIVE - Fear Composite | .17** | -.05 | .06 | -.14* | -.02 | .01 | .20** | -.14* | -.13* |
| 18. EPII – Positive Change | -.12* | -.00 | -.03 | .11 | .14* | -.07 | -.03 | .15* | .09 |
| 19. EPII – Negative Impacts | .24*** | -.07 | .13* | -.06 | -.06 | .19*** | .21*** | -.00 | -.09 |

**Table 2 continued.**

*Bivariate and Point-biserial Correlations Among Study Variables*

| Variable | 10 | 11 | 12 | 13 | 14 | 15 | 16 | 17 | 18 |
| --- | --- | --- | --- | --- | --- | --- | --- | --- | --- |
| 1. Gender |  |  |  |  |  |  |  |  |  |
| 2. Language |  |  |  |  |  |  |  |  |  |
| 3. Nativity |  |  |  |  |  |  |  |  |  |
| 4. Marital Status |  |  |  |  |  |  |  |  |  |
| 5. Education |  |  |  |  |  |  |  |  |  |
| 6. Essential |  |  |  |  |  |  |  |  |  |
| 7. Vulnerable |  |  |  |  |  |  |  |  |  |
| 8. Income |  |  |  |  |  |  |  |  |  |
| 9. Insurance |  |  |  |  |  |  |  |  |  |
| 10. GAD-7 | – |  |  |  |  |  |  |  |  |
| 11. PHQ-9 | .72*** | – |  |  |  |  |  |  |  |
| 12. PCL-5 | .76*** | .74*** | – |  |  |  |  |  |  |
| 13. FIVE - Contamination | .45*** | .28*** | .48*** | – |  |  |  |  |  |
| 14. FIVE - Social Distancing | .51*** | .43*** | .56*** | .64*** | – |  |  |  |  |
| 15. FIVE - Behaviors | .23*** | .09 | .24*** | .33*** | .27*** | – |  |  |  |
| 16. FIVE - Impact | .65*** | .46*** | .58*** | .53*** | .55*** | .26*** | – |  |  |
| 17. FIVE - Fear Composite | .54*** | .40*** | .58*** | .89*** | .92*** | .33*** | .60*** | – |  |
| 18. EPII - Positive Change | -.10 | -.27*** | -.04 | .17** | .04 | .23*** | -.02 | .10 | ­– |
| 19. EPII - Negative Impacts | .49*** | .46*** | .57*** | .28*** | .32*** | .20*** | .32*** | .34*** | .06 |

*Note*. Essential = Essential worker in the home. Vulnerable = Person vulnerable to COVID in the home. GAD-7 = Generalized Anxiety Disorder Scale, PHQ-9 = Patient Health Questionnaire, PCL-5 = PTSD Checklist, FIVE = Fear of Illness and Virus Evaluation, EPII = Epidemic Pandemic Impacts Inventory. Gender is coded 0=male, 1=female. Language is coded 0=Spanish, 1=English. Nativity is coded 0=foreign-born, 1=U.S.-born. Marital status is coded 0=single/divorced/widowed/separated, 1=married/living with partner. Essential worker is coded 0=no, 1=yes. Vulnerable is coded 0=no, 1=yes. Insurance is coded 0=no, 1=yes.

**p*<.05, ***p*<.01, ****p*<.001.
